# Emergence of new pathogens as a function of changes in host susceptibility.

**DOI:** 10.3201/eid0304.970404

**Published:** 1997

**Authors:** J G Morris, M Potter

**Affiliations:** University of Maryland School of Medicine, Baltimore 21201, USA. jmorris@umppa1.ab.umd.edu

## Abstract

A pathogen may emerge as an important public health problem because of changes in itself or its transmission pathways. Alternatively, a microorganism may emerge as a pathogen or acquire new public health importance because of changes in host susceptibility to infection. Factors influencing host susceptibility within the population as a whole include increases in the number of immunocompromised patients; increased use of immunosuppressive agents, particularly among persons receiving cancer chemotherapy or undergoing organ transplantation; aging of the population; and malnutrition. In considering the emergence of foodborne pathogens and designing interventions to limit their spread, the susceptibility of these population subgroups to specific infections should be taken into account.


### Emergence of New Pathogens as a Function of Changes in Host Susceptibility

**J. Glenn Morris, Jr.* and Morris Potter†**
*University of Maryland School of Medicine, Baltimore, Maryland, USA; and
†Centers for Disease Control and Prevention, Atlanta, Georgia, USA

A pathogen may emerge as an important public health problem because of changes in itself or its transmission pathways. Alternatively, a microorganism may emerge as a pathogen or acquire new public health importance because of changes in host susceptibility to infection. Factors influencing host susceptibility within the population as a whole include increases in the number of immunocompromised patients; increased use of immunosuppressive agents, particularly among persons receiving cancer chemotherapy or undergoing organ transplantation; aging of the population; and malnutrition. In considering the emergence of foodborne pathogens and designing interventions to limit their spread, the susceptibility of these population subgroups to specific infections should be taken into account.

Occurrence of disease is a function of several major variables: the virulence of the microorganism (i.e., its possession of factors that allow it to cause illness), its mode of transmission (i.e., how it gets to the host), and host susceptibility (i.e., how well the host can defend itself against the microorganism). Increased susceptibility to infection may be measured in terms of infectious dose (the number of microorganisms it takes to cause illness) and of the ability of the host to limit spread of the microorganism (e.g., the ability to limit spread of microorganisms from the intestinal tract to the bloodstream). These same variables apply to emerging pathogens. Microorganisms may emerge as pathogens because they have developed new virulence genes or resistance to standard therapeutic methods. Emergence may be related to changes in transmission pathways, which permit a known pathogen to move into new, previously unexposed populations. Finally, increases in the number of persons susceptible to a specific microorganism may result in its emergence as an important public health problem; at the same time, when attention is focused on populations with increased susceptibility to infection, organisms that might not otherwise be recognized as pathogens may be identified.

Address for correspondence: Glenn Morris, 934 MSTF, University of Maryland School of Medicine, 10 S. Pine St., Baltimore, MD, 21201, USA; fax: 410-706-4581; e-mail: jmorris@umppa1.ab.umd.edu.

Many factors influence the susceptibility of populations to infection, including increases in diseases that cause immunosuppression, increased use of immunosuppressive agents, aging of the population, and malnutrition. In considering these categories, it should be recognized that "host susceptibility" is not a single entity. Changes in host susceptibility may be due to various mechanisms, with each mechanism having a greater or lesser impact on the ability of the host to defend itself against infection with specific pathogens or classes of pathogens. These are very complex biologic systems; nonetheless, the general categories outlined below may be of value in identifying groups or populations for further study.

### Increases in Diseases That Cause Immunosuppression

Hereditary diseases associated with immunosuppression are present in a small but relatively constant proportion of the population. The most common of these diseases, a selective immunoglobulin A deficiency, has been found in as many as 0.3% of some blood donor populations (1) and may be associated with recurrent diarrhea; infections with giardia, in particular, have been noted to be more common among such immunocompromised patients.

In contrast to patients with hereditary immunodeficiencies, the population of patients with acquired immunodeficiencies is rapidly increasing. As of June 1996 in the United States, an estimated 223,000 persons ≥ 13 years of age had AIDS (Figure 1); this represents increases of 10% and 65% since mid-1995 and January 1993, respectively. During 1995, human immunodeficiency virus (HIV) infection remained the leading cause of death among persons 25 to 44 years of age (Figure 2), accounting for 19% of deaths from all causes in this age group (2).

Persons with AIDS show a clear increase in susceptibility to infection with *Salmonella* species. Data suggest that risk for nontyphi *Salmonella* infections is increased 20- to 100-fold among AIDS patients (3-7). Among persons infected with *Salmonella*, AIDS results in a severalfold increase in the risk for septicemia (3,5,6); AIDS also results in increases in infections at other extraintestinal sites, compatible with an overall increase in risk for dissemination of the organism. This increase in risk is reflected in increases in the percentage of total *Salmonella* isolated from blood. For example, for persons ages 25 to 49 years in states with high AIDS incidence, the percentage of *Salmonella* isolates from blood increased from 2.3% in 1978 to 17.8% in 1987 among men and from 3.1% to 8.1% among women; in contrast, no changes in blood-isolate percentages occurred for either sex in states with low AIDS incidence (8). These latter studies further suggest that serotype is an important risk determinant, with increases in bacteremia in states with high AIDS incidence associated primarily with infections due to *Salmonella* Enteritidis and *Salmonella* Typhimurium. While these data are for nontyphoidal *Salmonella*, studies outside the United States suggest that AIDS patients have a similar increase in risk for infection with *Salmonella typhi* in areas endemic for typhoid fever. For example, in Lima, Peru, the risk for typhoid increased 25-fold in HIV-infected persons 15 to 35 years of age (9); HIV infection also appeared to influence the clinical presentation of typhoid fever, with severe diarrhea and gastrointestinal symptoms seen more often than expected.

While the above data suggest a continuing climb in salmonellosis and, in particular, *Salmonella* bacteremia in conjunction with an increasing AIDS prevalence, anecdotal data from AIDS clinicians do not support this view. The standard of care for AIDS patients now includes routine prophylactic therapy with trimethoprim/sulfamethoxazole to prevent *Pneumocystis carinii* pneumonia. Trimethoprim/sulfamethoxazole is also one of the first-line therapies for salmonellosis; widespread prophylactic use of this drug in the AIDS population may have reduced the

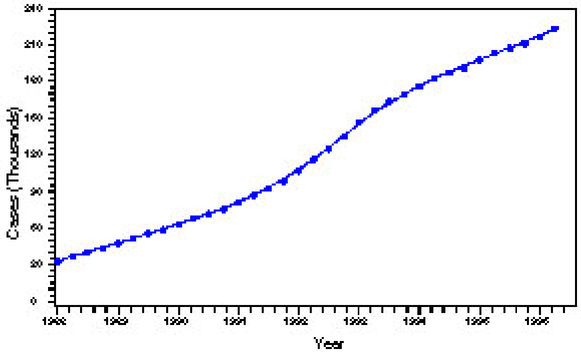

Figure 1. Cases among persons aged ≥ 13 years, adjusted for delays in reporting, by quarter year, United States, 1988–June 1996. (Points represent quarterly prevalence; the line represents "smoothed" prevalence. Estimates are not adjusted for incomplete reporting of diagnosed AIDS cases or AIDS deaths. From the Centers for Disease Control and Prevention. Update: Trends in AIDS incidence, deaths, and prevalence—United States, 1996. MMWR Morb Mortal Wkly Rep 1997;46:165-73).

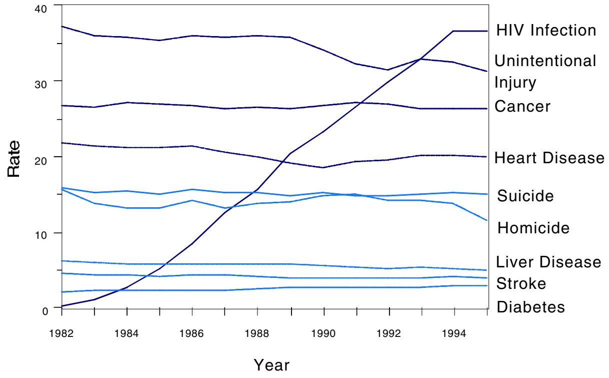

Figure 2. Death rates per 100,000 population for leading causes of death among persons ages 25 to 44 years, by year, United States, 1982–1995. (Based on underlying causes of death reported on death certificates, using final data for 1982–1994 and preliminary data for 1995. From the Centers for Disease Control and Prevention. Update: Trends in AIDS incidence, deaths, and prevalence, United States, 1996. MMWR Morb Mortal Wkly Rep 1997;46:165-73).

incidence of serious *Salmonella* infections, although this protective effect could be diminished in the face of increasing resistance to this antimicrobial agent among clinical isolates of *Salmonella* (10).

Fewer data are available on susceptibility of AIDS patients to other acute bacterial foodborne infections. While the initial impression was that the risk for *Campylobacter jejuni* infections was not higher among AIDS patients, a 35-fold increase in the *Campylobacter* case rate among persons with AIDS was noted in one study from Los Angeles (11). Other data indicate that HIV-positive patients can contract persistent *C. jejuni* infections, with chronic diarrhea, fever, and fecal leukocytes (12). In studies in the San Francisco area, AIDS patients were estimated to have a 280-fold increase in incidence of listeriosis, as compared with the general population (13). Of 98 nonpregnant adults with invasive *Listeria* infection identified between November 1988 and December 1990 in selected counties in California, Tennessee, Georgia, and Oklahoma, 20% were HIV-positive (13).

Before AIDS, *Toxoplasma gondii* was of concern primarily because of the risk for congenital infection in infants of mothers who had acute illness during pregnancy. *T. gondii* is now the leading cause of space-occupying cranial lesions in persons with AIDS (14,15); data from the 1980s suggest that 5% to 10% of AIDS patients get toxoplasmic encephalitis (16). In an estimated 50% of cases, *Toxoplasma* is transmitted by food (17). In this context, *Toxoplasma* must be regarded as an important emerging pathogen in this patient population.

The AIDS epidemic has also drawn attention to microorganisms not previously recognized as pathogens. Perhaps the most important of these is *Cryptosporidium*. In early investigations of AIDS-associated diarrhea, it rapidly became apparent that most patients were not infected with traditional enteric pathogens. Many of these patients were infected with *Cryptosporidium*; an estimated 10% to 20% of cases of AIDS-associated diarrhea are due to this microorganism (18). In subsequent years, *Cryptosporidium* was also recognized as the cause of intestinal infections in healthy hosts (19); and, most recently, it has been recognized as a major waterborne pathogen (20). *Isospora belli* has also been implicated as a cause of diarrhea in AIDS patients, as have the microsporidia (18). More recently, enteroaggregative *Escherichia coli* and nonpathogenic bioserovars of *Yersinia enterocolitica* were associated with diarrheal disease in AIDS cases (21,22). Further work will determine if these latter agents are indeed emerging pathogens in this patient population.

### Increased Use of Immunosuppressive Agents

Advances in medical treatment have resulted in increasing numbers of immunosuppressed patients (including patients undergoing organ transplantation or cancer chemotherapy) and patients with serious underlying chronic diseases; these patients, too, may be at increased risk for infection with microorganisms that might otherwise not be recognized or associated with serious illness. The number of new cancer cases has steadily increased over the past 20 years. For white males, cancers at all sites have increased from an estimated 364 new cases/100,000 population in 1973 to 462 new cases/100,000 population in 1994; white females show an increase from 295 new cases/100,000 population in 1973 to 347 new cases/100,000 population in 1994 (23). Patients are also surviving longer. For white males, the 5-year relative cancer survival rate was 56.6% in 1986 to 1993, compared with 42% in 1974 to 1976; for white females, the 1986 to 1993 rate was 62.3%, compared with 57.6% in 1974 to 1976. While specific data are lacking, these increases appear to have been accompanied by increased use of chemotherapeutic regimens and regimens that may have more toxicity than those used in the past. The number of solid organ transplants has also increased substantively (Figure 3). In particular, more complex procedures such as liver, heart, and lung transplants have increased. This, in turn, has resulted in larger numbers of chronically immunocompromised persons in the general population.

Aside from the direct immunosuppressive effect of the agents administered to these patients, other associated factors may contribute to susceptibility to infection. Many, if not most, patients receiving chemotherapy or immunosuppressive agents are also treated with antimicrobial drugs, which can have profound effects on the bacterial flora of the intestinal tract. These disturbances of gut microbial ecology may predispose to colonization and infection with other microorganisms, some of which may have increased virulence. Many chemotherapeutic

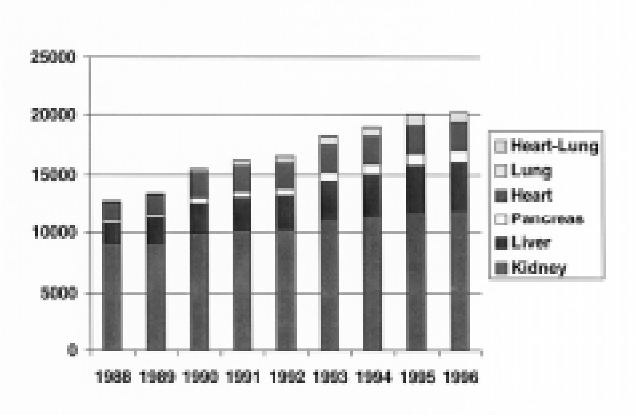

Figure 3. Number of organ transplants, by year and by site, 1988–1996 (data obtained from the United Network for Organ Sharing).
Note: Heart-lung transplants too few to be visible.

agents have direct toxicity for the gut mucosa; the resulting mucositis increases the susceptibility of these patients to bloodstream infections with whatever microorganisms are present in gut. The concentration of these highly susceptible patients on certain wards or units in a hospital may also increase the risk for nosocomial transfer of specific microorganisms. For example, vancomycin-resistant enterococci (VRE) (which, at least in Europe, have been associated with food [24]) have emerged as a substantive problem in cancer centers and transplant units (25-27). Persons who are immunosuppressed or have serious underlying illness are much more susceptible to colonization and infection with the organism. In some oncology and transplant units, more than 10% of patients are colonized or infected with VRE (26,27), providing well-documented opportunities for transfer of the organism to other immunosuppressed hosts in the same unit.

Cancer patients who have just undergone chemotherapy are often profoundly neutropenic. In this setting, especially in conjunction with the mucositis mentioned above, virtually any microorganism in the intestinal tract can enter the bloodstream and cause potentially fatal illness. For example, it has been found that raw produce in salads may be an important route by which patients acquire *Pseudomonas* (28); as a result, severely neutropenic patients are generally restricted to cooked food. *Salmonella* infections are reported among cancer patients, although the relative risk for infection in this population is not well characterized. Patients with neoplastic disease do appear to have a substantively increased risk for *Salmonella* septicemia, with 35% patients in one series having septicemia (29), versus fewer than 1% in healthy hosts. Cancer patients appear to be at increased risk for invasive *Listeria* infections (13). Toxoplasmosis tends to be of particular concern among transplant patients (30,31).

### Aging of the Population

The absolute number of the elderly in the United States is rapidly increasing, as is the proportion of the U.S. population they comprise. In 1950, 3.8 million persons (2.6% of the population) were over the age of 74, as opposed to 14.7 million (5.6% of the population) in 1995 (23; Figures 4 and 5). The elderly appear to be at a clearly increased risk for death from foodborne and diarrheal disease. Between 1979 and 1987, 28,538 persons in the United State had diarrhea as an immediate or underlying cause of death; 51% of these persons were more than 74 years of age, 27% were adults age 55 to 74, and 11% were children under the age of 5 (32).

The increased susceptibility of the elderly to infection and death may be due to a number of factors. Aging results in senescence of the gut-associated lymphoid tissue, increasing susceptibility to infection. Aging may also result in a decrease in gastric acid secretion: in one study, stimulated acid output was reduced approximately 30% in persons aged 65 to 98, with a 40% reduction in pepsin output (33). As a low pH of the

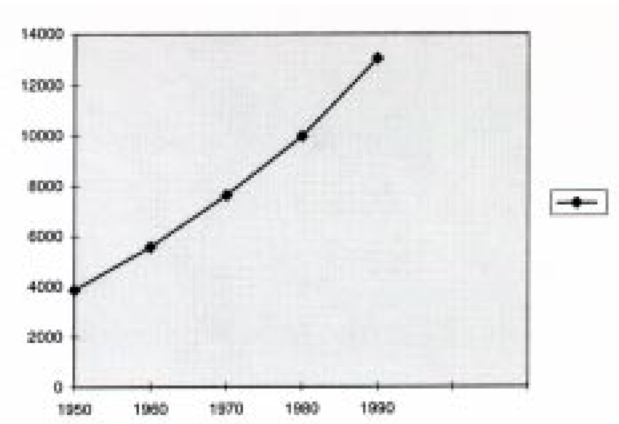

Figure 4. Number of persons >74 years of age, U.S. population, for selected years, 1950–1990. From the National Center for Health Statistics. Health, United States, 1996–97 and Injury Chartbook. Hyattsville, Maryland, 1997.

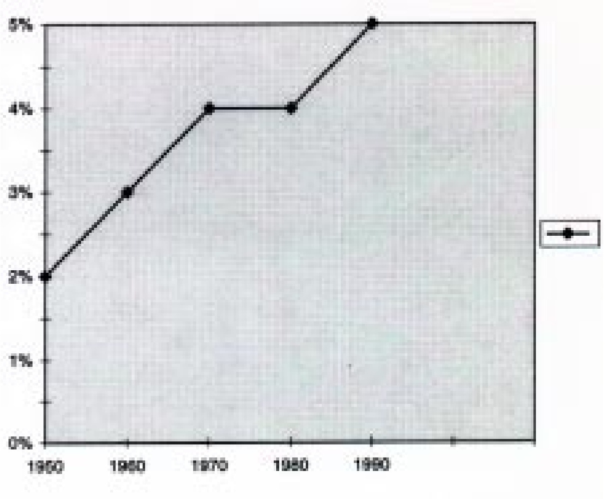

Figure 5. Percentage of U.S. population >74 years of age, for selected years, 1950–1990. From National Center for Health Statistics. Health, United States, 1996–97 and Injury Chartbook. Hyattsville, Maryland, 1997.

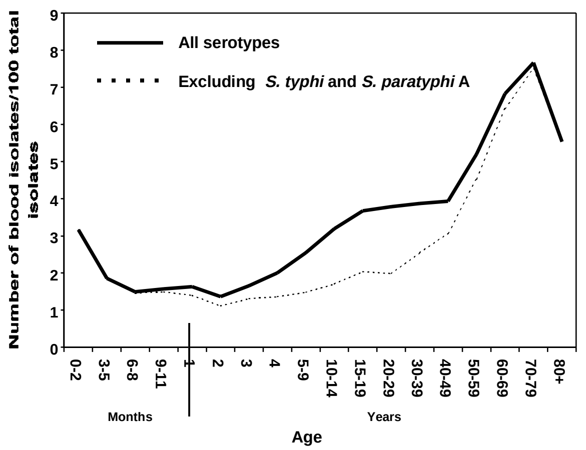

Figure 6. Ratio of blood isolates to total isolates of *Salmonella* by age group of the person from whom the isolate was obtained as reported to the Centers for Disease Control, Atlanta, GA, in 1968–79. From Blaser MJ, Feldman RA. Salmonella bacteremia: reports to the Centers for Disease Control and Prevention, 1968–1979. J Infect Dis 1981;143:743-6.

stomach is a major barrier to entry of enteric pathogens, reductions in gastric acidity can clearly increase the susceptibility to infection. Social factors may also influence susceptibility. For the elderly, residence in a long-term care facility was a major independent risk factor for diarrheal death (32). While a number of factors contribute to the increased risk, the communal living environment, combined with problems of fecal incontinence, create an environment in which enteric and foodborne pathogens are easily spread (32,34).

Incidence of salmonellosis and *Campylobacter* diarrhea appears to be higher among the elderly (35,36). More striking, however, is the increase in frequency of *Salmonella* bacteremia as compared with isolations from other sites: *Salmonella* infections in the elderly are more likely to cause bacteremia (37; Figure 6), which, in turn, substantively increases the risk for death. For example, in a recent nursing home outbreak in Maryland, 50 (35%) of 141 residents became ill, seven had bacteremia, nine were hospitalized (with a median length of hospitalization of 22 days), and four died (38). *E. coli* O157:H7 is also a common pathogen in nursing homes and among the elderly. In one reported nursing home outbreak, 55 of 169 residents were affected; overall, 19 (35%) of the affected residents died (39).

### Malnutrition

The above discussions have focused on issues most relevant to the United States and other industrialized countries. However, on a global scale, probably the leading cause of increased host susceptibility to infection is malnutrition. While accurate data on the prevalence of malnutrition are difficult to obtain, problems are accentuated in developing countries, in areas of political unrest, and among marginalized populations in the United States and other affluent nations. In Mexico, according to a probabilistic survey in 1990, 42.3% of children under 5 years of age had some degree of malnutrition (40).

Malnutrition increases host susceptibility through a number of mechanisms. It weakens epithelial integrity and may have a profound effect on cell-mediated immunity, with functional deficiencies in immunoglobulins and defects in phagocytosis. Malnutrition also may initiate a "vicious cycle" of infection predisposing to malnutrition and growth faltering, which in turn may lead to an increased risk for further infection (40,41). In studies in Bangladesh, malnourished and well-nourished children had the same number of infections with diarrheal pathogens such as enterotoxigenic *E. coli*; however, diarrhea in malnourished children was of longer duration and had greater potential long-term nutritional consequences (42). Overall, malnutrition appears to result in a 30-fold increase in the risk for diarrhea-associated death (40).

### Conclusions

Host susceptibility (and changes in the susceptibility to infection of groups within the general population) is a critical variable in assessing the public health effects and understanding the emergence and spread of pathogenic microorganisms. Surveillance within populations with increased susceptibility to infection may allow identification of new pathogens before they are recognized within the general population. Studies designed to identify the reasons for the increased susceptibility of a specific population to a specific agent may reveal how a microorganism is able (or not able) to breach normal host defense mechanisms. Finally, from a public health standpoint, risk management strategies for emergent foodborne pathogens must clearly identify and focus on populations with increased susceptibility to infection.

### References

1. Buckley RH. Primary immunodeficiency diseases. In: Bennett JC, Plum F, editors. Cecil textbook of medicine. Philadelphia: W.B. Saunders Company; 1996.p.1401-8.
2. Centers for Disease Control and Prevention. Update: trends in AIDS incidence, deaths, and prevalence—United States, 1996. MMWR Morb Mortal Wkly Rep 1997;46:165-73.
3. Celum CL, Chaisson RE, Rutherford GW, Barnhart JL, Echenberg DF. Incidence of Salmonellosis in patients with AIDS. J Infect Dis 1987;156:996-1002.
4. Smith PD, Macher AM, Bookman MA, Boccia RV, Steis RG, Gill V, et al. *Salmonella typhimurium* enteritis and bacteremia in the acquired immunodeficiency syndrome. Ann Intern Med 1985;102:207-9.
5. Profeta S, Forrester C, Eng RHK, Liu R, Johnson E, Palinkas R, Smith SM. Salmonella infections in patients with acquired immunodeficiency syndrome. Arch Intern Med 1985;145:670-2.
6. Gruenewald R, Blum S, Chan J. Relationship between human immunodeficiency virus infection and Salmonellosis in 20- to 59-year-old residents of New York City. Clin Infect Dis 1994;18:358-63.
7. Angulo FJ, Swerdlow DL. Bacterial enteric infections in persons infected with human immunodeficiency virus. Clin Infect Dis 1995;21:S84–93.
8. Levine WC, Buehler JW, Bean NH, Tauxe RV. Epidemiology of nontyphoidal *Salmonella* bacteremia during the human immunodeficiency virus epidemic. J Infect Dis 1991;164:81-7.
9. Gotuzzo E, Frisancho O, Sanchez J, Liendo G, Carrillo C, Black RE, Morris JG Jr. Association between the acquired immunodeficiency syndrome and infection with *Salmonella typhi* or *Salmonella paratyphi* in an endemic typhoid area. Arch Intern Med 1991;151:381-2.
10. Lee LA, Puhr ND, Maloney EK, Bean NH, Tauxe RV. Increase in antimicrobial-resistant *Salmonella* infections in the United States, 1989–1990. J Infect Dis 1994;170:128–34.
11. Sorvillo FJ, Lieb LE, Waterman SH. Incidence of campylobacteriosis among patients with AIDS in Los Angeles County. J Acquir Immune Defic Syndr Hum Retrovirol 1991;4:598-602.
12. Perlman DM, Ampel NM, Schifman RB, Cohn DL, Patton CM, Aguirre ML, et al. Persistent *Campylobacter jejuni* infections in patients infected with human immunodeficiency virus (HIV). Ann Intern Med 1988;108:540-6.
13. Schuchat A, Deaver KA, Wenger JD, Plikaytis BD, Mascola L, Pinner RW, et al. Role of foods in sporadic listeriosis. 1. Case-control study of dietary risk factors. JAMA 1992;267:2041-50.
14. Luft BJ, Brooks RG, Conley FK, McCabe RE, Remington JS. Toxoplasmid encephalitis in patients with acquired immune deficiency syndrome. JAMA 1984;252:913-7.
15. Porter SB, Sande MA. Toxoplasmosis of the central nervous system in the acquired immunodeficiency syndrome. N Engl J Med 1992;327:1643-8.
16. McCabe RE, Remington JS. *Toxoplasma gondii*. In: Mandell GL, Douglas RG, Bennett JE, editors. Principles and practice of infectious diseases. 3rd ed. New York: Churchill Livingston; 1990. p. 2090-103.
17. United States Department of Agriculture. Pathogen reduction; hazard analysis and critical control point (HACCP) systems; proposed rule. Washington (DC): Federal Register 1995;60:6774-889.
18. Smith PD, Quinn TC, Strober W, Janoff EN, Masur H. Gastrointestinal infections in AIDS. Ann Intern Med 1992;116:63-77.
19. Wolfson JS, Richter JM, Waldron MA, Weber DJ, McCarthy DM, Hopkins CC. Cryptosporidiosis in immunocompetent patients. N Engl J Med 1985;312:1278-82.
20. Centers for Disease Control and Prevention. Assessing the public health threat associated with waterborne cryptosporidiosis: report of a workshop. MMWR Morb Mortal Wkly Rep 1995;44(RR-6):19.
21. Mayer HB, Acheson D, Wanke CA. Enteroaggregative *Escherichia coli* are a potential cause of persistent diarrhea in adult HIV patients in the United States. In: Program and abstracts of the 31st US-Japan Cholera and Related Diarrheal Diseases Conference; 1995 Dec 1-3; Kiawah Island, South Carolina.
22. Saillour M, de Truchis P, Risbourg M, Nordman P, Nauciel C, Perronne C. *Yersinia enterocolitica* gastroenteritis in HIV infected patients. In: Abstracts of the 35th Interscience Conference on Antimicrobial Agents and Chemotherapy; 1995 Sept 17-20; San Francisco, California.
23. National Center for Health Statistics. Health, United States, 1996-97 and Injury Chartbook. Hyattsville (MD): The Center; 1997.
24. Bates J, Jordens JZ, Griffiths DT. Farm animals as a putative reservoir for vancomycin-resistant enterococcal infection in man. J Antimicrob Chemother 1994;34:507-16.

25. Morris JG Jr, Shay DK, Hebden JN, McCarter RJ, Perdue BE, Jarvis W, et al. Enterococci resistant to multiple antimicrobial agents, including vancomycin: establishment of endemicity in a university medical center. Ann Intern Med 1995;123:250-9.
26. Papanicolaou GA, Meyers BR, Meyers J, Mendelson MH, Lou W, Emre S, et al. Nosocomial infections with vancomycin-resistant *Enterococcus faecium* in liver transplant recipients: risk factors for acquisition and mortality. Clin Infect Dis 1996;23:760-6.
27. Montecalvo MA, Horowitz H, Gedris C, Carbonaro C, Tenover FC, Issah A, et al. Outbreak of vancomycin-, ampicillin-, and aminoglycoside-resistant *Enterococcus faecium* bacteremia in an adult oncology unit. Antimicrob Agents Chemother 1994;38:1363-7.
28. Remington JS, Schimpff SC. Please don't eat the salads. N Engl J Med 1981;304:433-5.
29. Wolfe MS, Armstrong D, Louria DB, Blevins A. Salmonellosis in patients with neoplastic disease. Arch Intern Med 1971;128:546-54.
30. Luft BJ, Naot Y, Araujo FG, Stinson EB, Remington JS. Primary and reactivated toxoplasma infection in patients with cardiac transplants. Ann Intern Med 1983;99:27-31.
31. Hofflin JM, Potasman I, Baldwin JC, Oyer PE, Stinson EB, Remington JS. Infectious complications in heart transplant recipients receiving cyclosporine and corticosteroids. Ann Intern Med 1987;106:209-16.
32. Lew JF, Glass RI, Gangarosa RE, Cohen IP, Bern C, Moe CL. Diarrheal deaths in the United States, 1979-87. JAMA 1991;265:3280-4.
33. Feldman M, Cryer B, McArthur KE, Huet BA, Lee E. Effects of aging and gastritis on gastric acid and pepsin secretion in humans: a prospective study. Gastroenterology 1996;110:1043-52.
34. Bennett RG. Diarrhea among residents of long-term care facilities. Infect Control Hosp Epidemiol 1993;14:397-404.
35. Hargrett-Bean N, Pavia AT, Tauxe RV. *Salmonella* isolates from humans in the United States, 1982-1986. MMWR Morb Mortal Wkly Rep 1988;30(SS-2):25-31.
36. Tauxe RV, Hargrett-Bean N, Patton CM, Wachsmuth K. *Campylobacter* isolates in the United States, 1982-1986. MMWR Morb Mortal Wkly Rep 1988;37(SS-2):1-13.
37. Blaser MJ, Feldman RA. Salmonella bacteremia: reports to the Centers for Disease Control, 1968-1979. J Infect Dis 1981;143:743-6.
38. Taylor JL, Dwyer DM, Groves C, Bailowitz A, Tilghman D, Kim V, et al. Simultaneous outbreak of *Salmonella enteritidis* and *Salmonella schwarzengrund* in a nursing home: association of *S. enteritidis* with bacteremia and hospitalization. J Infect Dis 1993;167:781-2.
39. Carter AO, Borczyk AA, Carlson JAK, Harvey B, Hogkin JC, Karmali MA, et al. A severe outbreak of *Escherichia coli* O157:H7-associated hemorrhagic colitis in a nursing home. N Engl J Med 1987;317:1496-500.
40. Santos JI. Nutrition, infection, and immunocompetence. Inf Dis Clin North Am 1994;8:243-67.
41. Black RE, Brown KH, Becker S. Effects of diarrhea associated with specific enteropathogens on the growth of children in rural Bangladesh. Pediatrics 1984;73:799-805.
42. Black RE, Brown KH, Becker S. Malnutrition is a determining factor in diarrheal duration, but not incidence, among young children in a longitudinal study in rural Bangladesh. Am J Clin Nutr 1984;37:87-94.
